# Children Strategically Decide What to Practice

**DOI:** 10.1111/cdev.14268

**Published:** 2025-05-31

**Authors:** Daniil Serko, Julia Leonard, Azzurra Ruggeri

**Affiliations:** ^1^ Technical University of Munich Munich Germany; ^2^ Yale University New Haven Connecticut USA; ^3^ Central European University Vienna Austria

**Keywords:** active learning, adaptation, preparedness, study‐effort allocation

## Abstract

Adjusting practice to different goals and characteristics is key to learning, but its development remains unclear. Across 2 preregistered experiments, 190 4‐to‐8‐year‐olds (106 female; mostly White; data collection: December 2021–September 2022) and 31 adults played an easy and a difficult game, then chose one to practice before a test on either the easy, difficult, or a randomly chosen game. All children adjusted their active practice choices to condition. When the test game was known, they practiced that game. However, when the test game was randomly chosen, only children 6+ and adults practiced the difficult game, while younger children only showed a trending effect. This suggests that the ability to prepare for uncertainty may develop between ages 4 and 6.

Imagine that you are back in primary school, and every Friday you get either a math or a French test. On some weeks, the teacher tells you in advance which subject you will be tested on. On the weeks you know you will get a math test, you focus on studying math, and on the weeks you know you will get a French quiz, you focus on studying French. However, on some other weeks, the teacher does not tell you in advance which subject you will be tested on. How should you prepare? The answer depends on your skill set. Given an unknown upcoming test, you should strategically decide to practice the subject that is harder for you to minimize your chances of failing. If you are really good at math and confident that you will be able to do well on a math test without studying very much, you should focus on studying French. However, if you are really good at French, you might want to focus on studying math. In other words, strategic practice choices depend on both the goal (e.g., what test you need to prepare for) and the task characteristics (e.g., what task is more difficult for you).

Although a primary school Math or French quiz may seem trivial, decisions about what and how we practice build up over time to determine what we learn and who we become. This idea aligns with Ericsson's notion of deliberate practice, which posits that repeated actions aimed at improving a specific skill are crucial for achieving high performance (Ericsson et al. 1993). Practice involves the deliberate allocation of attention and efforts towards specific tasks or goals, incorporating a range of cognitive and behavioral processes. These processes include executive function, prospective memory and future thinking, metacognition and self‐monitoring (Ericsson 2008; Ericsson and Harwell 2019; Ericsson et al. 1993, 2009). However, while for Ericsson “practice” refers to gradual improvement through the repetition of an action (Ericsson 2008; Ericsson and Harwell 2019; Ericsson et al. 1993, 2009), the example above specifically refers to a flavor of practice that is more strategic and active. In this paper, we focus on what we term “ecological active practice”—the strategic choices students make regarding how to invest their time and effort in preparing for the future. This concept aligns more closely with the literature on active and self‐directed learning than with traditional educational views of practice. In this context, we situate our concept within the ecological active learning framework (see Ruggeri 2022). Ecological active learning involves actively exploring and learning by recognizing and leveraging the specific structure and features of a learning task or environment. Learners adapt their exploratory and learning strategies to maximize efficiency and effectiveness, taking into account task goals, characteristics, available resources, and their own prior knowledge and abilities. Ecological active practice, in turn, refers to a strategic, personalized approach in which students make deliberate choices about how to invest their time and effort to prepare for future challenges.

Considering the critical role of ecological active practice in learning, it is surprising how little we know about its developmental trajectory. Gaining insight into its development is essential, not only for informing theoretical discussions about the relative advantages of active versus instructed learning across different stages of development (Bonawitz et al. 2011; Gureckis and Markant 2012; Klahr and Nigam 2004; Piaget 1930), but also for practically understanding when and how to best scaffold children's learning to help them develop into competent and independent adults. Here, we explore whether children aged 4 to 8 are able to adapt their practice choices based on the goals (e.g., which task they will be tested on) and task characteristics (e.g., task difficulty) to maximize rewards and minimize losses.

Prior research suggests that adults adapt their practice choices appropriately based on both goals and task characteristics (Baranes et al. 2014; Kornell and Metcalfe 2006; Locke and Latham 2002; Metcalfe 2011; Metcalfe and Kornell 2005; O'Doherty et al. 2017; Ten et al. 2021). For example, Ten et al. (2021) presented participants with games that varied in difficulty and told them to play for a given number of trials. With no external constraints, adults spent their time playing easier games, on which they made fast progress (see also, Baranes et al. 2014). However, when participants were instructed to learn all games because they would eventually be tested on them, they were more likely to spend their time playing more difficult games (Ten et al. 2021). Similarly, when preparing for a test of novel word pairs, adults studied items of intermediate difficulty and avoided spending time studying items they already knew or that were very difficult (Metcalfe and Kornell 2005). In short, adults effectively chose to practice more difficult items before a test. Given the maturity of practice decisions in the adult state, we turn our attention to when in development children possess this capacity.

Prior developmental work paints a contradictory picture of whether and in what situations children possess the cognitive capacities to strategically engage in active practice (Brinums et al. 2018; Casey and Redshaw 2022; Cimpian 2017; Davis et al. 2016; Flavell et al. 1970; Magid et al. 2018; Metcalfe and Finn 2013; Wang and Bonawitz 2022). On the one hand, research on decision‐making and metacognition suggests that, unlike adults and older children, younger children's practice choices are not adaptive, in that they do not systematically take into account goals and task characteristics (Brinums et al. 2018, 2021; Casey and Redshaw 2022; Flavell et al. 1970; Metcalfe and Finn 2013). For example, preschoolers and third graders do not allocate more study time to a memory test item they previously did poorly on (Flavell et al. 1970; Metcalfe and Finn 2013), and it is not until fifth grade (i.e., around 10 years of age) that children begin to behave like adults, devoting extra study time to yet‐to‐be‐learned items before a test (Metcalfe and Finn 2013). Furthermore, when 4‐ to 7‐year‐old children are encouraged to practice one of three games before a test, only 6‐ and 7‐year‐old children strategically practice the game that they will be tested on (Brinums et al. 2018). Overall, this line of work suggests that the ability to tailor one's practice strategies to the goals and characteristics of a given task may develop rather late in childhood, between ages 6 and 10.

On the other hand, a growing body of research with younger children on persistence, exploration, and information search provides compelling evidence that even toddlers can make adaptive learning choices in response to goals and task characteristics (Bridgers et al. 2020; Davis et al. 2016; Leonard et al. 2017, 2020; Lucca et al. 2020; Magid et al. 2018; Ruggeri 2022; Ruggeri et al. 2019; Rule et al. 2023; Wang and Bonawitz 2022). Infants and children put more effort into a task when evidence suggests it is difficult (e.g., they observe an adult who needed to put effort into succeeding Leonard et al. (2017, 2020) and Lucca et al. (2020)). Toddlers are more persistent in their search when there is more information to be gathered with their actions (Ruggeri et al. 2023) and adapt their search strategy to the task characteristics to maximize their information gain (Ruggeri et al. 2017, 2019). Studies have shown that when the goal is to play for fun rather than to win, children aged 5 to 10 are more likely to choose a more challenging version of a game (Rule et al. 2023). Also, in simpler, more constrained and controlled paradigms, even 4‐year‐olds demonstrate forward‐thinking by selecting objects that help them solve future tasks (Suddendorf and Moore 2011) and by choosing to practice the game they know they will later be tested in a forced‐choice task, despite being unable to explicitly say why practice is important (Davis et al. 2016). This line of research suggests that, by the preschool years, children are already able to adapt their actions based on specific goals and in response to task characteristics, such as its difficulty and information structure.

One key difference between the two lines of work discussed above is the targeted age range, which influences the task designs and paradigms used. Specifically, research on metacognition and decision‐making typically tests children aged 4 to 11 and often involves paradigms that rely heavily on verbal instructions and impose high memory demands. These tasks sometimes require children to follow multi‐step procedures and remember specific rules (e.g., Brinums et al. 2018; Casey and Redshaw 2022; Flavell et al. 1970; Metcalfe and Finn 2013), which may disadvantage younger children.

This raises the possibility that selective active practice abilities may emerge in children earlier than previously thought when presented with minimally demanding paradigms. However, beyond task demands, two key gaps remain in the literature. First, no prior work has explicitly manipulated both task characteristics (e.g., task difficulty) and goals (e.g., what to prepare for) within the same paradigm in children. As a result, it remains unclear whether and when children can use *both* factors to inform their *practice* choices. Second, no prior work has explored children's active practice choices in the face of uncertainty. This is crucial to examine, as it provides valuable insights into how children develop decision‐making skills, navigate uncertain situations while minimizing potential losses, and maximize learning opportunities and efficiency.

Importantly, prior work suggests that the ability to prepare for mutually exclusive possibilities emerges at age 4 (Davis et al. 2016; Redshaw et al. 2018), suggesting that even young children can reason about uncertain situations (Coughlin et al. 2015; Ghetti et al. 2013; Goupil et al. 2016; Hembacher and Ghetti 2014; Lyons and Ghetti 2013; Redshaw et al. 2018; Suddendorf et al. 2017). Thus, it is possible that even preschool‐age children may use their understanding of alternative future outcomes to strategically decide what to practice.

Here, we set out to answer whether children make ecological active practice choices. In particular, across two preregistered experiments (see OSF‐links below), we investigated 4‐ to 8‐year‐old children's and adults' ability to tailor their active practice choices to the goals (i.e., preparing for a known or unknown test) and characteristics of a given task (i.e., the difficulty of the task). This relatively wide age range was strategically selected to bridge the two lines of research reviewed above. By testing children across this age range, we aimed to capture both the emergence and refinement of active practice, which may be influenced by developmental changes in metacognitive abilities (e.g., Fleur et al. 2021; Whitebread and Neale 2020), executive functions (e.g., Best and Miller 2010; Lee et al. 2014), and information‐search abilities (e.g., Poli et al. 2024; Ruggeri 2022). Additionally, we included an adult sample to provide a performance benchmark.

We specifically designed our experiments to be engaging and understandable for children across the age range targeted. Specifically, we used child‐friendly games (guess the picture and block building), employed memory aids, ample visual examples, and many comprehension check questions to make sure children understood our procedure (see 1.1 Methods and 2.1 Methods).

Experiment 1 implemented a within‐subjects design where children (4‐ to 8‐year‐old) and adults played an online guessing game in which they had to guess pictures of familiar animals and objects that were partially occluded. Experiment 2 implemented a between‐subjects design where children (4‐ to 5‐year‐old) played a hands‐on, minimally verbal brick‐building task. In both studies participants were familiarized with an easy and a difficult version of a game and were then informed about a later test in which they would be presented with either the easy (*Test‐Easy* condition), the difficult (*Test‐Difficult* condition), or a randomly chosen game (*Test‐Random* condition). Before entering the test, children were asked which of the two versions of the game they would like to practice.

We predicted that participants would make ecological active practice choices, tailored to the goal structure and task difficulty in both experiments with the aim to improve performance on future tests. Specifically, we hypothesized that participants would choose to practice the easy game in the *Test‐Easy* condition and the difficult game in the *Test‐Difficult* condition. Our critical condition of interest was the *Test‐Random* condition: we hypothesized that participants would choose to practice the difficult task in the *Test‐Random* condition to minimize potential losses. Indeed, they should expect to do relatively well on the easy test even without practice, but to perform poorly in the difficult test if they had not practiced it (see the hypothesis rationale section in the Supporting Information for more details). We tested a large age range in Experiment 1 to explore *when* children make adaptive active practice choices. In Experiment 2, we specifically targeted preschool‐age children to see if even young children could make adaptive active practice choices with very minimal task demands. Sample sizes for both studies were calculated using a priori power analysis on a simulated data set (see 1.1 Methods and 1.2 Method sections; Supporting Information). Preregistration, data, and analyses are available on the Open Science Framework (Overall project: https://tinyurl.com/4bj4dfb3; Experiment 1: https://tinyurl.com/33xptwus; Experiment 2: https://tinyurl.com/364tzkv9). Note that Study 1 in OSF corresponds to the pilot of Experiment 1 in this paper, while Study 2 in OSF corresponds to Experiment 1 in this paper.

## 1 Experiment 1

### 1.1 Methods

#### 1.1.1 Participants

Participants in Experiment 1 were 115 four‐ to eight‐year‐old children (64 females; *M* = 76.97 months; SD = 17.29 months; range: 48 to 107 months) and 31 adults (23 females, *M* = 29.42 years; SD = 10.25 years; range: 19 to 70 years). No ethnic or socio‐economic status data were collected, but the population from which the sample was drawn is approximately 71% ethnic German, 11% other European, 9% Middle Eastern, 3% Asian, 2% Afro‐German or Black African, and 4% other or unspecified, and encompasses a wide range of socio‐economic backgrounds. We recruited participants from the database of the Max Planck Institute for Human Development in Berlin (Germany) and the Technical University of Munich (Germany). Eight additional children were excluded from the analyses because they did not want to participate in the games (*n* = 2), they were outside our age target (*n* = 3), because of a technical malfunction (*n* = 1) or missing demographic data (*n* = 2). Additionally, participants' data were excluded on rounds in which they failed to answer a comprehension check question (*n* = 7 rounds) which in some analyses leads to a small deviation in reported total trials. We also tested one additional adult, who had to be excluded from the analyses because the equipment failed to record the session.

The study was approved by the IRB of the Max Planck Institute for Human Development in Berlin (Germany). Prior to the beginning of the experimental session, adults and children's parents signed an informed consent form online, and we asked children to give verbal consent to participate. To estimate the sample size prior to data collection, we performed a power analysis on a simulated dataset (see Supporting Information), which indicated that at least 80 children in total had to be tested to detect a difference between conditions with 80% power and 0.8 estimated effect size, with a 0.05 criterion for statistical significance. We tested more children than suggested by the power analysis to ensure an even age distribution within the sample.

#### 1.1.2 Design

**FIGURE 1:**
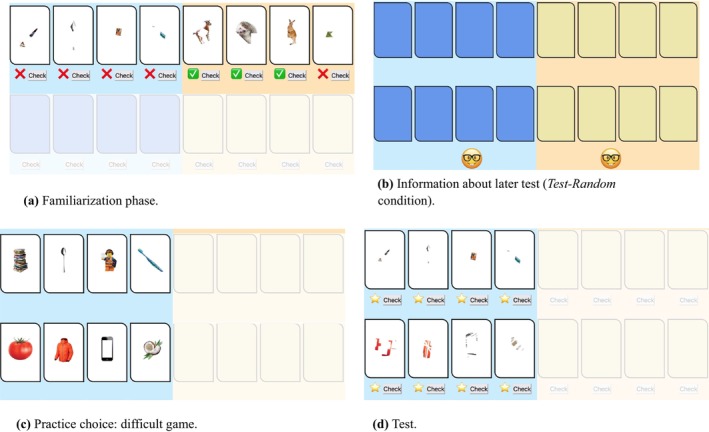
Screenshots from Experiment 1. (a) Children were presented with two games, where they had to identify partially‐occluded pictures of animals or objects (counterbalanced across participants). They were first familiarized with the games by playing the top four cards of each game. The easy game (in this example presented to the right) included three very easy‐to‐guess (80% visible) pictures and one difficult‐to‐guess picture (20% picture visible), whereas the difficult game (in this example presented to the left; sides were counterbalanced across participants) included four very difficult‐to‐guess pictures. During the familiarization rounds, the feedback (green tick for a correct answer vs. red cross for an incorrect answer) was displayed under the corresponding cards. The games were *not* explicitly labeled as easy or difficult by the experimenter to avoid biasing children. After the familiarization phase, we told children that they would eventually be tested on the easy game (*Test‐Easy* condition), on the difficult game (*Test‐Difficult* condition), or on a randomly chosen game (*Test‐Random* condition; conditions manipulated within subjects in counterbalanced order). In the *Test‐Easy* and *Test‐Difficult* condition, an emoji below the game they would be tested on provided a memory aid for children. (b) In the *Test‐Random* condition, the emoji was placed below both games, and the experimenter emphasized that for the time being they could not know in which of the two games they would eventually be tested. We then asked the children to decide which of the two games they wanted to practice before being tested. (c) Practice setup, where children practiced all the 8 images of the game they selected (the difficult game in this example). (d) Test setup, in which children were tested on all 8 images of the game they were assigned to (in this example, the difficult game; note that half children in the *Test‐Random* condition were eventually tested on the easy game, whereas the other half were eventually tested in the difficult game). At test, a golden or black star below an image indicated a correct or incorrect guess. For each correct answer in the tests children received one sticker, for each incorrect answer they lost one sticker.

Experiment 1 consisted of three rounds, across which we manipulated the conditions (*Test‐Easy*, *Test‐Difficult*, *Test‐Random*) within subjects (order of presentation counterbalanced). Each round included a familiarization, a practice, and a test phase (see Figure 1). We ensured through extensive pilot testing (*N* = 146) that the instructions, materials, and goals of the task were understandable for young children. Pilot data suggested that all children know the animals and objects used as stimuli. The games were designed using HTML, CSS, and JavaScript and are available online (https://daniilserko.de/follow_up/openlink.html). We conducted the experiment online using the Webex conferencing tool. Webex is an online conferencing tool similar to Zoom. Webex is a secure video conferencing platform that meets the data protection standards required by the authors' research organization. Children were seated next to their parents while the experimenter shared their screen. Before beginning the procedure, the experimenter confirmed via the parent that the stimuli were clearly visible to the children and that the procedure was appropriately displayed in full‐screen mode. Parents were reminded not to interfere with their children's responses during the experiment. Note that a meta‐analysis by Chuey et al. (2022) found that effect sizes in developmental studies conducted online were comparable to those in in‐person studies, underscoring the reliability of our online testing methodology.

##### 1.1.2.1 Familiarization Phase

The experimenter introduced participants to two guessing games: an *Animal*‐pictures game (8 cards) on one side of the screen, and an *Object*‐pictures game (8 cards) on the other side (side counterbalanced). Participants had to guess what the first 4 pictures (top row) of each game represented and received visual feedback on their performance (green tick for a correct answer, red cross for an incorrect answer), but were neither told the correct names for unknown items nor given explicit feedback about the difficulty of the games (see Figure 1a; Supporting Information; see also Lyons and Ghetti 2011 who used a similar approach). All the pictures showed animals and objects that participants were familiar with (e.g., a cat, a dog, a slice of pizza), but we varied the degree to which each picture was actually visible by covering part of the picture with white geometrical shapes: The easy game included three very easy‐to‐guess (80% visible) pictures and one difficult‐to‐guess picture (20% picture visible), whereas the difficult game included only very difficult‐to‐guess pictures.

We added one difficult‐to‐guess picture to the easy game, so that (i) it would be necessary to practice the easy game in the *Test‐Easy* condition to be able to guess all items correctly at test, and (ii) children would still perceive the easy game as challenging and fun (see Serko et al. 2022). To confirm that the easy and difficult games differed in difficulty as intended, we compared children's performance in both games during familiarization (see also the detailed analysis of children's performance in each game during familiarization and at test in the Supporting Information). As expected, in the large majority of easy‐game trials children were able to correctly guess 3/4 items (87%; 294 out of 338 trials), whereas only in a few difficult‐game trials children were able to correctly guess at least one of the items (4%; 15 out of 338 trials; for details see Supporting Information). A paired *t*‐test revealed a significant difference in performance between the easy and difficult game (*t*(337) = 126.10, *p* < 0.001, 95% CI [2.84, 2.93]).

##### 1.1.2.2 Practice Phase

After the familiarization phase, we provided participants with information about the test phase. In the *Test‐Easy* and *Test‐Difficult* conditions, we informed participants that they would eventually be tested on all eight cards of the easy or the difficult game, respectively. During this explanation of the test phase, we visually highlighted the side of the screen corresponding to the game they would be tested on by placing a smiley below the game and partially dimming the other side of the screen.

In the *Test‐Random* condition, we told participants that we would test them on a randomly chosen game. To emphasize this, the experimenter showed participants two cards that matched the colors of the cards in the two games. After shuffling the cards, the experimenter explained that she would randomly draw one of the cards, which would later indicate the target test game. The experimenter then randomly drew one of the cards without looking at it and without revealing it to the participant and set it aside. In order to further emphasize the uncertainty about the later test, we included a smiley icon beneath each game, highlighting the fact that the specific game for testing was not yet determined (see Figure 1b).

We told child participants that they would win one sticker for each correct answer and loose one sticker for each incorrect answer. We told adult participants that the highest performers would enter a lottery over a 50e Amazon voucher. We informed participants that they could choose to practice one of the two games before the final test. Specifically, the experimenter said: “Before you take the test, you can choose one of the two games that you would like to practice again. You can practice the [color of the game] game [point to the left side], in which you were [depending on game difficulty: already pretty good vs. not very good], or you can practice the [color of the game] game [point to the right side], in which you were [depending on game difficulty: already pretty good vs. not very good]. Which game would you like to practice again before taking the test?” Once participants made their choice, they entered the practice phase in which they again guessed the four practice cards and the four new cards (8 cards in total) of the chosen game and received corrective feedback (see Figure 1c).

##### 1.1.2.3 Test Phase

At test, participants had to guess all eight cards of the easy or difficult game in the *Test‐Easy* and *Test‐Difficult* conditions, respectively (see Figure 1d). In the *Test‐Random* condition, all participants were tested on the difficult game.

### 1.2 Results

#### 1.2.1 Adults' Active Practice Choices

As predicted, adults effectively adapted their active practice choices to the task goals and characteristics: A logistic mixed‐effects model predicting adults' active practice choice (easy or difficult game) by condition (*Test‐Easy*, *Test‐Difficult*, *Test‐Random*; *Test‐Easy* as baseline) as a fixed effect and participants' ID as a random effect revealed main effects of the *Test‐Difficult* condition (*p* = 0.002, OR = 0.100 [0.024–0.417]) and the *Test‐Random* condition (*p <* 0.001, OR = 0.055 [0.011–0.266]). Exploratory follow‐up analyses revealed that, as expected, only 26% of the adults in the *Test‐Easy* condition (*n* = 8/31) selected to practice the difficult game, which is significantly lower than chance (one‐tailed 50% binomial test, *p* = 0.005). In the *Test‐Difficult* and *Test‐Random* conditions, 71% (*n* = 22/31) and 81% (*n* = 25/31) of the adult participants, respectively, chose to practice the difficult game. Both proportions were significantly greater than chance (one‐tailed 50% binomial test, *Test‐Difficult* condition: *p* = 0.015; *Test‐Random* condition: *p <* 0.001). A chi‐square test revealed no difference in active practice choice between the *Test‐Difficult* and *Test‐Random* conditions (*χ* ^2^(1) = 0.352, *p* = 0.553) indicating that in both conditions participants made similar active practice choices. Thus, as predicted, we found that adults chose to practice the difficult games more often in the *Test‐Difficult* and *Test‐Random* conditions compared to the *Test‐Easy* condition.

#### 1.2.2 Children's Active Practice Choices

Overall, we found that children—like adults—effectively adapted their active practice choices to fit the goals and task characteristics. A logistic mixed‐effects model predicting children's active practice choices (easy or difficult) with condition (*Test‐Easy*, *Test‐Difficult*, *Test‐Random*; *Test‐Easy* as baseline) and age in months as fixed effects and participants' ID as a random effect revealed main effects of the *Test‐Difficult* condition (*p <* 0.001, OR = 0.197 [0.102–0.380]) the *Test‐Random* condition (*p <* 0.001, OR = 0.226 [0.119–0.430]). Exploratory follow‐up analyses revealed that, as expected in the *Test‐Easy* condition, 39% of the children (*n* = 44/113) selected the difficult game (significantly lower than chance, *p* = 0.012, one‐tailed 50% binomial test). As expected, in the *Test‐Difficult* condition and *Test‐Random* condition, 71% (*n* = 79/111) and 68% (*n* = 78/114) of the children, respectively, selected the difficult game. Both proportions were significantly greater than chance (one‐tailed 50% binomial test, *Test‐Difficult* condition: *p <* 0.001; *Test‐Random* condition: *p <* 0.001). A chi‐square test revealed no difference in task choice between the *Test‐Difficult* and *Test‐Random* conditions (*χ* ^2^(1) = 0.092, *p* = 0.761) indicating that in both conditions children made similar active practice choices. The analysis also revealed a main effect of age in months (*p <* 0.001, OR = 0.966 [0.949–0.983]), indicating that older children were more likely to choose to practice the difficult game.

**FIGURE 2:**
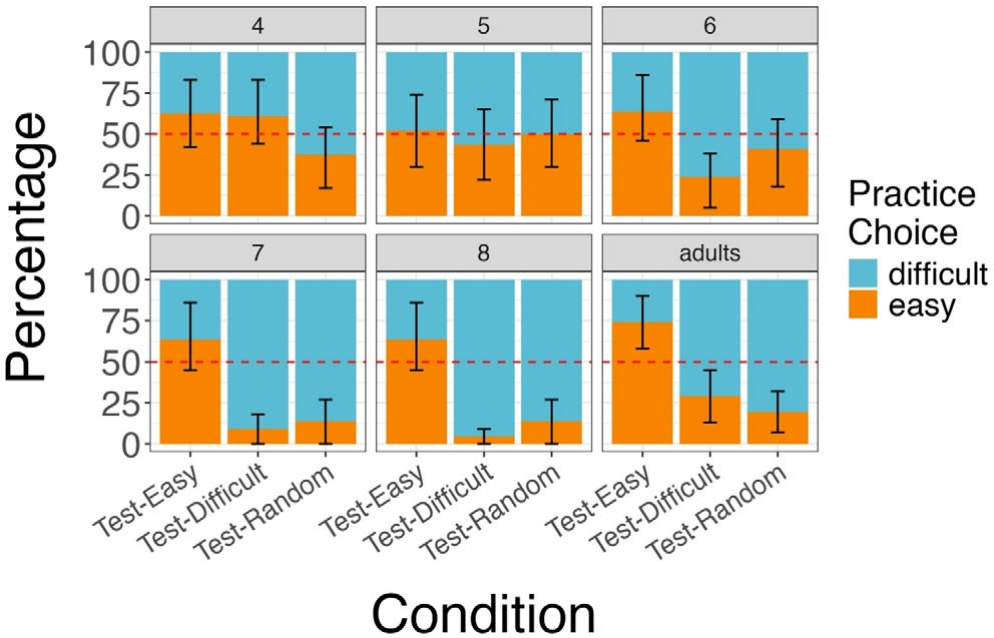
Illustration of participants' active practice choices by condition (*Test‐Easy*, *Test‐Difficult*, and *Test‐Random*) in Experiment 1 faceted by age in years (adults as a separate group). Bars represent the percentage of easy (blue) and difficult (orange) active practice choices for each condition. Error bars indicate 95% confidence intervals.

**FIGURE 3:**
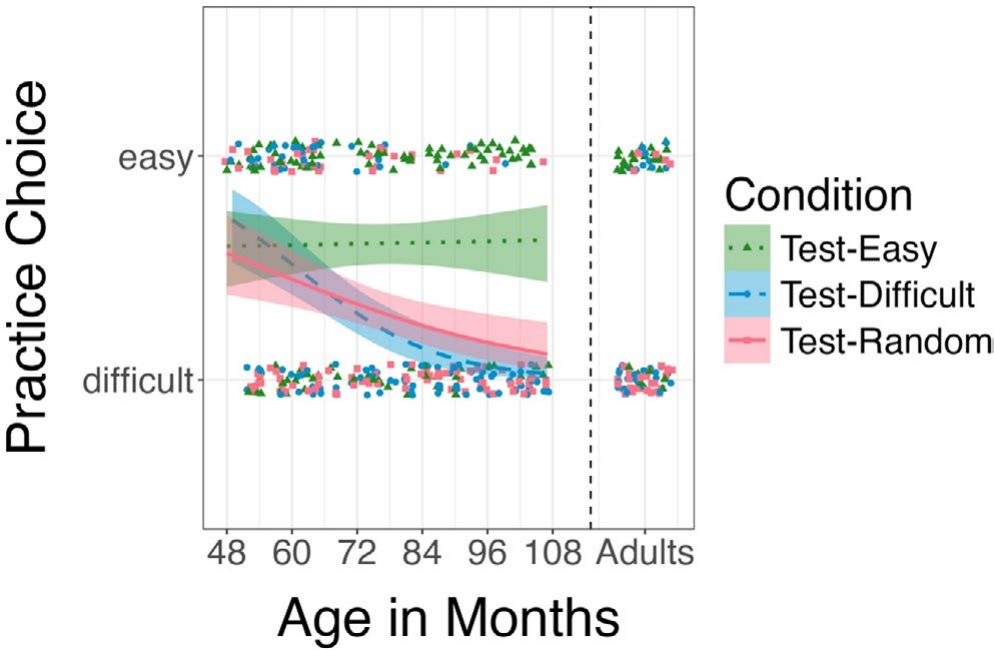
Illustration of participants' active practice choices, displayed by age (in months, with adults as a separated group on the right) and condition. Dots indicate individual active practice choices in a single game. The three lines are fitted logistic regressions with a 95% confidence interval by condition.

To further explore age effects, we conducted an exploratory logistic mixed‐effects model, predicting children's active practice choices by condition, age in months, and their interaction as fixed effects, and participants' ID as a random effect. The analysis revealed a significant interaction of the *Test‐Difficult* condition and age (*p <* 0.001, OR = 0.204 [0.092–0.453]), and the *Test‐Random* condition and age (*p* = 0.013, OR = 0.440 [0.231–0.838] see Figures 2 and 3), indicating that older children were more likely to practice the difficult game in these conditions. Note that this interaction model fits the data better (Akaike Information Criterion (AIC): 397.0) compared to the main effects model presented above (and preregistered; AIC: 412.7), without the interaction. In an additional model, we added round as a main effect predictor to investigate whether children's performance improved over the course of the experiment (i.e., as they played more rounds). However, adding round as a predictor did not improve the model fit (AIC: 414.6) and round did not contribute to predicting children's practice choices.

Finally, we performed an exploratory analysis to identify the age at which children begin making ecological active practice choices. Specifically, we compared the number of children within each age group, binned by years, who made adaptive active practice choices with the number of children who did not, using a one‐sample proportions test. We found that among 4‐ (54.93%; 39 out of 71 trials) and 5‐year‐old (52.86%; 37 out of 70 trials) children, performance was not significantly different from chance (two‐tailed 50% binomial test, *p* = 0.202 for 4‐year‐old children; *p* = 0.360 for 5‐year‐old children). By age 6, the majority of children (66%; *n* = 43 out of *n* = 65 trials) were able to make adaptive practice choices (two‐tailed 50% binomial test, *p* = 0.013; see Figure 2; Supporting Information for a complete breakdown of adaptive practice choices across age groups).

#### 1.2.3 Interim Summary

The findings from Experiment 1 indicate that both adults and children aged 6 and above make active practice choices that adapt to the goals and characteristics of a given task:

They choose to practice the task that they will be eventually tested on, and importantly, when they do not know which task they will be tested on, choose to study the more difficult task to make up for their potential losses. For the 4‐ and 5‐year‐old children, performance did not significantly differ from chance, leaving it unclear whether their active practice choices were intentional or merely random (see Figure 2; Supporting Information).

There are several reasons why younger children may have had difficulties adapting their active practice choices to the task goals and characteristics.

First, although we aimed to minimize linguistic and cognitive demands, we may not have reduced them sufficiently to accommodate the needs of younger children. The game relied on remembering the names of the objects learned during the practice round, which may have been much more challenging for younger children, whose memory is still developing (Gathercole 1998; McCormack and Atance 2011). Indeed, additional analyses of children's performance indicate that younger children, compared to older children, had difficulties with guessing the difficult items at test even when they had practiced them, indicating that these items were difficult to remember for them (see Supporting Information for details). The study design also required children to understand the concept of “randomness.” In Experiment 1, we illustrated randomness by shuffling in front of the children two cards, each representing the easy or the difficult game. The experimenter then told children that, at test, they would pick one of the cards and find out which game they would be tested on (see 1.1 Methods). Despite this, it is possible that children may have failed to understand that they had a 50% chance of being tested in either the easy or the difficult game. Furthermore, we did not implement a comprehension check assessing whether children understood the given goal of the task (see Locke and Latham 2002). Thus, it could be the case that younger children chose what to practice based on their own goals (e.g., perhaps to maximize their fun), rather than the given task goals. Finally, the experimental session in Experiment 1 took about 20 min to complete, which may have been too long for young children.

## 2 Experiment 2

Experiment 2 aimed to test whether 4‐ and 5‐year‐old children can make adaptive active practice choices when presented with a less demanding version of the paradigm used in Experiment 1. To address the limitations mentioned above, we made several changes to the paradigm. First, we made the task more child‐friendly and less cognitively demanding by using a brick‐building task that did not rely on verbal memory and allowed children to indicate their choices by pointing. We also conducted the tests in person to ensure maximum engagement. Additionally, we provided explicit feedback regarding the difficulty level of each game, labeling them as “easy” or “difficult” after children had familiarized themselves with each game. Furthermore, we explicitly demonstrated the concept of randomness to young children by blindly picking between a box with easy‐to‐stack or difficult‐to‐stack blocks and confirmed that children understood this manipulation (see Design for details). We also implemented a series of comprehension checks to make sure children fully understood the goal and the characteristics of their assigned condition. We predicted that with these extra scaffolds, even 4‐ and 5‐year‐old children would make adaptive active practice choices. Finally, we changed our paradigm to be between‐subjects, which reduced the average length of the experimental session from 20 to 6 min, and reduced cognitive demands by only presenting children with one condition.

### 2.1 Methods

#### 2.1.1 Participants

Participants were 75 (*n* = 25 per condition) 4‐ to 5‐year‐old children (42 females, *M* = 59.72 months; SD = 6.79 months; range: 48 to 71 months). We recruited and tested participants in the public Zoo of Berlin (Germany). No ethnic or socio‐economic status data were collected, but the population from which we obtained the sample was the same as in Experiment 1. We recruited 36 additional children (22 females, *M* = 56.09 months; SD = 7.87 months; range: 37 to 71 months), but excluded them from further analysis due to preregistered exclusion criteria: failure to answer the comprehension questions correctly (*n* = 13), difficulties or failure to understand the instructions or to perform the brick building task (*n* = 9), experimenter error (*n* = 4), because they were too shy to interact with the experimenter (*n* = 3), they were outside our age range (*n* = 3), because of technical failure (*n* = 3), or because they watched another child's active practice‐choice making (*n* = 1).

We acknowledge a high exclusion rate in our study, which we attribute to several factors. Beyond the typical challenges of working with young children—such as shyness, distraction, or inattentiveness—two additional factors likely contributed. First, Experiment 2 was conducted at the zoo, providing strong ecological validity by placing children in an engaging, educational environment. However, the lively setting, with exotic animals and other visitors, also made it harder for children to concentrate on the tasks and follow instructions. As a result, the higher exclusion rate for failing comprehension checks and struggling with the block task may stem from the distractions inherent to this stimulating environment.

Second, we implemented different comprehension‐check questions to ensure that all included children understood the task instructions. If a participant failed a comprehension check, the experimenter corrected them and repeated the question. Participants were excluded from the analysis if they failed to answer correctly after three attempts. Specifically, before beginning the main task, we asked children to: (1) identify which of the two games was easier and which was more difficult for them, (2) confirm whether they knew the blocks they would use during the test (*Test‐Easy* and *Test‐Difficult* conditions) or did not know them (*Test‐Random* condition), (3) confirm that the goal was to build the tallest tower to win stickers, and (4) verify that they understood they had a limited number of attempts during the test phase.

Consequently, we excluded 6 participants for incorrectly identifying the game difficulty and 7 participants for failing to accurately indicate their knowledge about the game they would later be tested on. While this exclusion process was stringent, we believe it was essential to maintain the integrity of our results, ensuring a more accurate understanding of the developmental trajectory in children's active practice choices. Also, note that only 13 participants were excluded due to failing a comprehension check questions, while the remaining 23 exclusions were due to factors such as difficulty building the towers during familiarization, shyness in interacting with the experimenter, age ineligibility, experimenter error, or observing another child's practice choice.

The sample size was determined by conducting a simulation‐based a priori power calculation to detect the hypothesized effect with 80% power and a 0.4 difference between the *Test‐Easy* condition on one side and the *Test‐Random* condition on the other side, with a 0.05 criterion for statistical significance. The difference of 0.4 between conditions was based on the results of Experiment 1. This sample‐size calculation indicated a sample of 50. Given that we have a third condition (*Test‐Difficult* condition), we tested 25 additional children, for a total of 75 children.

Before beginning the experimental session, parents signed an informed consent, and children were asked to give verbal consent to participate. The study was approved by the ethics committee of the Max Planck Institute for Human Development in Berlin (Germany).

#### 2.1.2 Design

**FIGURE 4:**
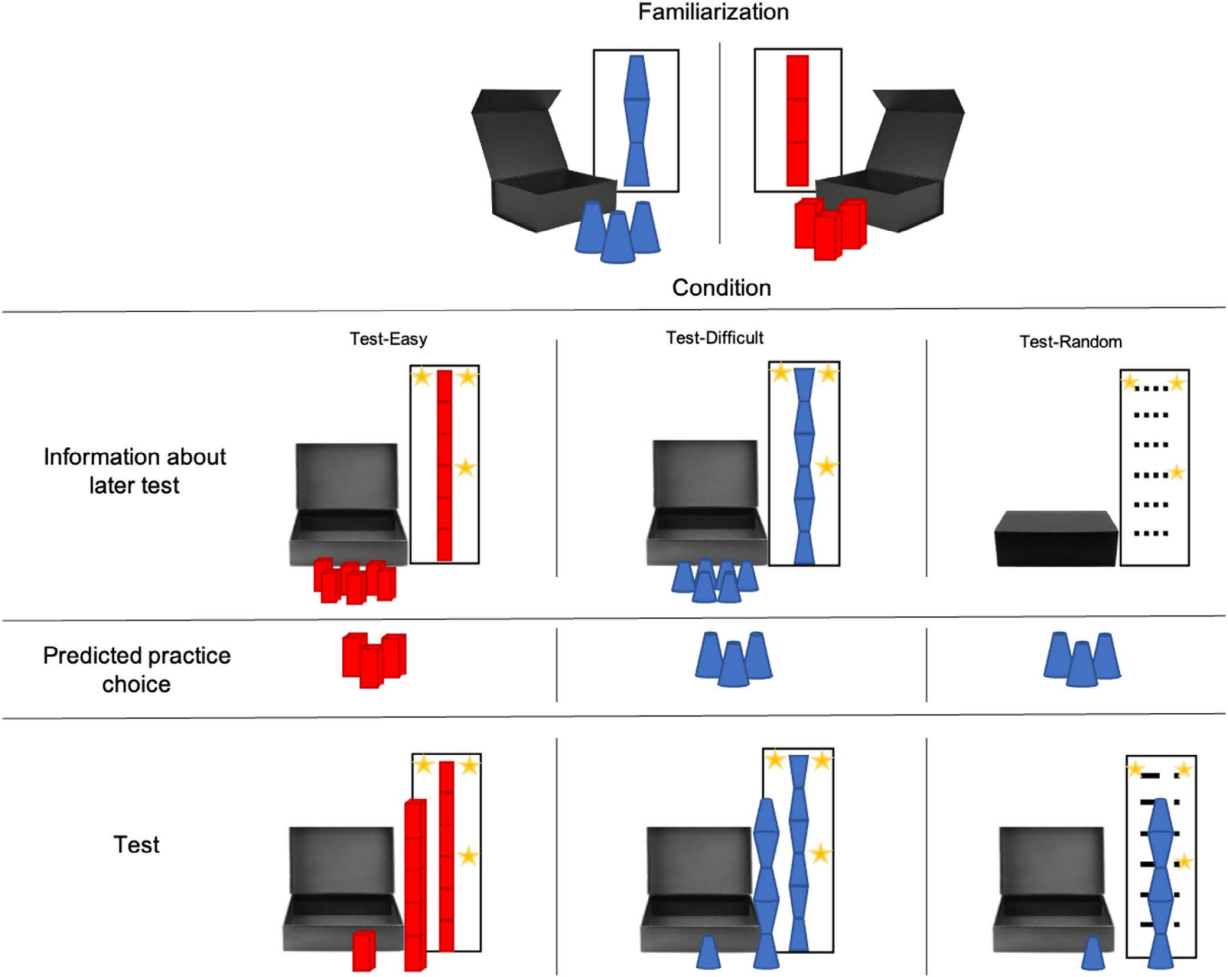
Illustration of the procedure of Experiment 2. Children were presented with two games, in which they had to construct towers using blocks. They first familiarized themselves with each game. The easy game (in this example presented on the right) featured three easy‐to‐assemble cuboid blocks, while the difficult game (in this example presented on the left; sides and colors were counterbalanced across participants) included three oddly shaped and difficult‐to‐build blocks. The order and color of the two games were counterbalanced across participants. After the familiarization phase, we told children that they would eventually be tested on the easy game (*Test‐Easy* condition), on the difficult game (*Test‐Difficult* condition), or on a randomly chosen game (*Test‐Random* condition), and that they could win stickers depending on the height of the tower they built. Children were told that if they managed to build a tower out of three blocks at test, they would win one sticker; if they managed to build a tower out of six blocks, they would win two stickers (indicated with stars). If the tower collapsed, children would not win anything. We then asked the children to decide which of the two games they wanted to practice before being tested. At test, children in the *Test‐Easy* condition were given 6 easy blocks. Children in the *Test‐Difficult* condition were given 6 difficult blocks. In the *Test‐Random* condition, children were given a randomly chosen box containing 6 blocks of either the easy or difficult‐to‐stack blocks. A golden star on the level of three stacked blocks indicated that children won 1 sticker; two golden stars on the level of 6 stacked blocks indicated that children won 2 stickers.

Children played two building‐block games: In the easy game, they had to build a tower with three easy‐to‐staple cuboid blocks, whereas in the difficult game, they had to build a tower of three oddly‐shaped, difficult‐to‐staple blocks (color and order were counterbalanced across participants; see Figure 4; see scripts in the Supporting Information). After a set of comprehension checks (see familiarization phase below), we informed the children that we would eventually test them on the easy game (*Test‐Easy* condition), the difficult game (*Test‐Difficult* condition), or a randomly chosen game (*Test‐Random* condition), and that they could win stickers depending on the height of the tower they built: one sticker for a three‐block tower, two stickers for a six‐block tower. However, if the tower collapsed, children would not win anything. Children could then choose which of the two games they wanted to practice before entering the test phase.

We ensured through extensive pilot testing (*N* = 125) that our instructions were clear, and that young children could perform the tasks. All materials used in the experiment were novel and specifically built for the experiment. To ensure that building a tower with regular blocks was indeed easier than with oddly‐shaped blocks, we measured how long it took children to build a tower with each set of blocks. To avoid a bias towards larger objects, the blocks used in both games were built to have the exact same height (63 mm; for details see Supporting Information).

##### 2.1.2.1 Familiarization Phase

Children sat on a blanket next to the experimenter in front of two covered identical boxes. The experimenter uncovered one of the boxes and introduced the children to three identical blocks and a picture of a three‐block tower built out of the three blocks, which children had to reproduce using the given blocks. The experimenter then repeated the same procedure with the other box. In the easy game, the blocks were easy‐to‐stack regular cuboid blocks; in the difficult game, the blocks were difficult‐to‐stack oddly shaped blocks (see Figure 4; Supporting Information).

After the children successfully built each tower, the experimenter gave explicit feedback on the difficulty of the two games (“wow that was easy” or “wow that was difficult”) and asked whether they believed they could get better with practice at that particular game. Irrespective of their response, the experimenter always said that children could get better at each game with practice. After this, we asked children to indicate which game was easy and which one was difficult for them. If they failed to answer correctly (*n* = 14), we asked them to rebuild both towers and repeated the comprehension check question. If they again failed to answer correctly (*n* = 6), they were excluded from the study. Children faced no time restriction to build each tower, and we included only children in the sample who managed to build both towers (*n* = 2; excluded for failing to build the difficult tower). The order of presentation of the games (easy or difficult), as well as the color of the blocks (blue or red) was counterbalanced. As expected, during familiarization, children needed significantly less time to build the three brick tower with the easy blocks (*M* = 8.47 s, SD = 4.38) compared to building the difficult tower (*M* = 20.58 s, SD = 16.58). A Welch two‐sample *t*‐test revealed a significant difference in performance between the easy and difficult game (*t*(81.985) = −6.036, *p <* 0.001).

##### 2.1.2.2 Practice Phase

Children were randomly assigned to one of three conditions: *Test‐Easy*, *Test‐Difficult*, and *Test‐Random*. In the *Test‐Easy* and the *Test‐Difficult* conditions, children were presented with an open box (covered until then) containing six blocks of the same color and shape as those presented in the easy or difficult game, respectively (see Information about later test in Figure 4). Next to the box, there was a picture illustrating a tower made out of six of those blocks: at the three‐block height, there was one star and at the six‐block height, there were two stars indicating that children would win 1 or 2 stickers depending on their performance. We informed children that, at test, they would have to build a tower out of the blocks in that box to win stickers: they would win one sticker if they managed to build a three‐block tower, and two stickers for a six‐block tower.

In the *Test‐Random* condition, children were presented with two *closed* identical boxes (covered until then). The experimenter opened the two boxes, one by one, and showed that they contained either the easy‐to‐staple or the difficult‐to‐staple blocks (order counterbalanced). The experimenter then closed both boxes, began shuffling them behind a curtain with her eyes closed, and explained to the children that at the count of three she would pick one random box with her eyes closed, which she did. Pilot testing indicated that when the experimenter kept her eyes closed during the procedure (as opposed to keeping them open), children clearly understood that she was selecting a box at random, rather than intentionally choosing a specific box for them to be tested on. The closed box was placed next to the illustration showing the required tower height for children to construct during the testing phase (see Figure 4). Children in all conditions were told that at test they would have only one shot at building the tower, and that if the tower collapsed they would not win anything.

Before entering the test phase, we asked children which one of the two games they wanted to practice in order to prepare for the test. This choice was our main dependent variable. Children then practiced with the three blocks of the chosen game until they managed to build a three‐block tower (see Figure 4).

##### 2.1.2.3 Test Phase

At test, children were given the box with the test blocks and were asked to build a six‐block tower. The results indicated that children who encountered the easier blocks, on average, built significantly taller towers (*M* = 5.83 blocks, SD = 0.65) compared to those who were presented with the difficult blocks (*M* = 4.43 blocks, SD = 1.34; *t*(29.832) = 4.602, *p <* 0.001, Welch two‐sample *t*‐test).

### 2.2 Results

#### 2.2.1 Practice Choices

**FIGURE 5:**
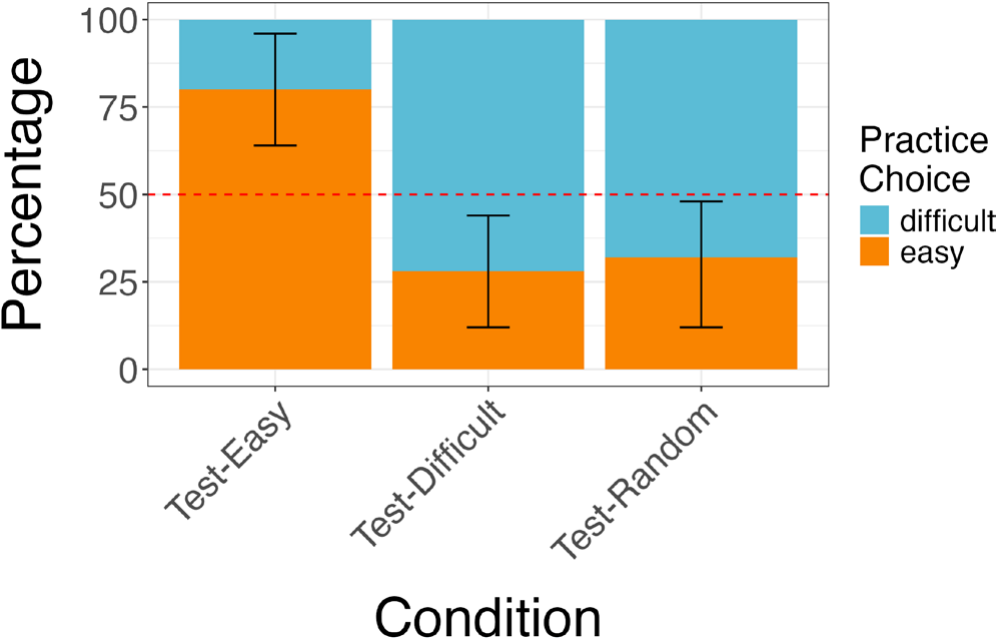
Illustration of participants' active practice choices by condition in Experiment 2. Each bar represents one of three conditions: *Test‐Easy*, *Test‐Difficult*, or *Test‐Random*. The *y*‐axis shows the percentage of participants' choices between the easy and difficult games. The blue color indicates that participants chose to practice the difficult game, while the orange color indicates participants chose to practice the easy game. The red dotted line indicates the 50% chance level. The error bars extending above and below the bars represent the 95% confidence intervals.

The analyses yielded mixed findings on whether 4‐ to 5‐year‐old children effectively adapted their active practice choices to the task goals and characteristics (see Figure 5). A logistic regression model predicting children's active practice choices (easy or difficult) with condition (*Test‐Easy*, *Test‐Difficult*, *Test‐Random*; *Test‐Easy* as baseline) revealed main effects of the *Test‐Difficult* condition (*p <* 0.001, OR = 0.097, [0.024–0.339]) and the *Test‐Random* condition (*p <* 0.001, OR = 0.118, [0.030–0.403]). An exploratory analysis adding age in months to the model revealed that the reported main effects of conditions hold and indicated no significant effect of age. In the *Test‐Easy* condition, only 20% of the children (*n* = 5/25) selected the difficult game. This proportion was significantly lower than chance (*p* = 0.002, one‐tailed 50% binomial test). In the *Test‐Difficult* condition, 72% (*n* = 18/25) of the children selected the difficult game. This proportion was significantly greater than chance (*p* = 0.022, one‐tailed binomial test). In the *Test‐Random* condition, 68% (*n* = 17/25) of the children selected the difficult game. While this proportion was fairly close to the proportion of the *Test‐Difficult* condition, it was trending to be significantly greater than chance (*p* = 0.054, one‐tailed binomial test), although it did not meet the conventional significance level. The CIs of the *Test‐Random* condition do not overlap the 50% chance rate, which further suggests an effect but would require further investigation (see Figure 5). A chi‐square test revealed no difference in task choice between the *Test‐Difficult* and *Test‐Random* conditions (*χ* ^2^(1) = 0.000, *p* = 1.000), indicating that in both conditions, children made similar active practice choices. Therefore, 4‐ to 5‐year‐old children adapt their practice strategies to different goals (chi‐square tests of condition difference), but they do not consistently choose to practice the difficult task above chance in the random condition, though there is a trend towards significance (binomial regression for the *Test‐Random* condition).

## 3 Discussion

We find that 4‐ to 8‐year‐old children are capable of making adaptive, ecological active practice choices that align with adult‐like strategic behavior. Specifically, when children know which task they will be tested on, they selectively practice that task. Importantly, when the task is uncertain, 6‐ to 8‐year‐olds tend to practice the more difficult one. Preschool‐age children show a similar pattern, though the effect is only trending toward significance, suggesting that the ability to strategically choose a harder task in preparation for an uncertain future may emerge between ages 4 and 6.

In Experiment 1, we found evidence of developmental changes in children's ecological active practice choices. In particular, children did not begin to selectively practice the game they would soon be tested on until age 6. Moreover, it was not until age 7 that children behaved like adults, choosing to practice the more difficult game when unsure which game they would be tested on. This finding is consistent with research on the developmental trajectory of question asking by Ruggeri and Lombrozo (2015) and Ruggeri et al. (2017, 2019), which indicates that at ages 7 and 10, children adapt their strategies as readily as adults, even though their baseline performance differs.

In Experiment 2, we found that even preschool‐age children can adapt their active practice choices to different goals when presented with a simplified task and provided with adequate scaffolding. However, although the majority of 4‐ and 5‐year‐olds chose to practice the difficult game in the random condition, this effect did not reach statistical significance. It is possible that our study was just underpowered, and a larger sample might reveal more robust evidence for this effect. Alternatively, the ability to strategically select an appropriate training task in preparation for an uncertain future may emerge between ages 4 and 6. Nevertheless, the fact that children adjusted their practice choices based on the condition is noteworthy. Prior research has been inconclusive about whether young children can flexibly adapt their practice strategies, and our findings provide new evidence that even preschool‐age children begin to show systematic, goal‐directed adjustments in their practice behavior (Brinums et al. 2018; Cimpian 2017; Magid et al. 2018; Metcalfe and Finn 2013; Wang and Bonawitz 2022).

Why did preschool‐age children show some adaptation in their practice choices in Experiment 2 but not as clearly in Experiment 1? One possibility is that Experiment 2 had lower task demands compared to Experiment 1. Specifically, Experiment 2 was substantially shorter and less complicated than Experiment 1 because it employed a between‐subjects design with a child‐friendly block‐building task. Additionally, Experiment 2 was conducted in person (unlike Experiment 1, which was online), with explicit labeling of game difficulty and a clear demonstration of randomness—where the experimenter selected a box blindfolded behind a curtain. We also ensured comprehension by including only children who passed a series of checks. Any of these factors could have contributed to the children's success. Future research is needed to pinpoint which elements are most critical in helping young children adapt their practice choices effectively.

More broadly, our research adds to the ongoing discourse about whether and how self‐directed, active learning enhances learning depth and quality when contrasted with instructed learning (Bruner et al. 1976; Kuhn 2000; Montessori 1912/1964; Piaget 1930). In particular, recent work shows that allowing children and adults to actively control their learning experience (e.g., decide what to study, in what order and for how long) improves their memory of the learned materials (Chi 2009; Gureckis and Markant 2012; Markant and Gureckis 2014; Markant et al. 2016; Ruggeri et al. 2019). While our findings indicate that even young children can make active learning choices that prepare them for the uncertainty of the future, we acknowledge that our study does not investigate the efficiency of these decisions compared to instructed learning decisions in terms of their impact on learning outcomes. Future research should directly compare children's learning outcomes in active learning settings with those in instructed learning environments to draw more explicit conclusions about the effects of active versus instructed learning on children's learning processes.

Our findings also contribute to debates concerning young children's *wishful thinking* (Bernard et al. 2016; Lipko et al. 2009; Schneider 1998; Wente et al. 2019). For example, Wente et al. (2019) demonstrated that 3‐ to 5‐year‐old children were likely to overestimate the occurrence of a low‐probability event (like drawing a rare card) when they were promised a reward upon the event's occurrence, compared to when the reward was guaranteed regardless. This finding suggests that young children's predictions are often biased by their desires. However, if the participants in our study had been primarily motivated by wishful thinking, they should have expected in the *Test‐Random* condition to be tested in the easy game (which was way less likely to result in a disappointing failure, and offered the perspective of winning more stickers), and therefore should have practiced the easy game. However, we find that the large majority of children in the *Test‐Random* condition chose to practice the difficult game. Thus, our results suggest that children do not always let their desires control their beliefs about the future and therefore bias their actions aimed to prepare for an unknown future state. This is interesting also in light of recent evidence suggesting that preschoolers struggle to align their actions to the probability of future events (Crimston et al. 2023).

While our results offer promising insights and potential for future research avenues, we also recognize a few limitations of our experiments. First of all, our participant pool was predominantly comprised of European children. This might reduce the generalizability and therefore the broader applicability of our findings to other cultural contexts. Future studies should prioritize the inclusion of a more diverse sample and control for variability in socioeconomic status (SES) and parental education. Second, the current results do not allow us to monitor individual differences leading to performance variation. Future work should identify specific individual factors, including cognitive competencies, which may mediate children's promptness and competence to make adaptive active practice choices. Third, our experimental design was relatively narrow, centered around a single task (either guessing item names or building a tower) presented at two varying difficulty levels (easy and difficult versions of a game). However, the realm of active practice choices encompasses a much wider spectrum of complexity. A promising avenue for future research would explore children's active practice choices within and across different domains and types of tasks—for instance, deciding to practice a physical task versus delving into a cognitive task. Fourth, we used comprehension check questions to ensure that all children understood the task. These checks were designed to confirm a basic understanding, not to select only the most attentive or advanced participants. Future research could explore varying the complexity of these checks to better assess how different levels of attentiveness or cognitive ability impact children's active practice choices. Fifth, we provided explicit feedback to participants in order to control their inferences about task difficulty. Future work should explore both how children represent and reason about their own abilities in the absence of performance feedback and whether they can use these internal assessments to prepare for future unknowns. Sixth, since our design did not initially aim to examine 4‐ and 5‐year‐olds separately, we combined their data in Experiment 2. While this allowed us to examine broader patterns, it limited our ability to capture finer developmental differences within this age range. Future research with larger samples could help disentangle these age‐related changes, offering a more detailed understanding of early developmental trends.

The decisions individuals make about what to practice and learn accumulate over the course of their lives. Eventually, these active practice choices end up shaping people's competencies and expertise, their likelihood “*to be well prepared*” for whatever may come—in the words of a 4‐year‐old girl explaining why she chose to practice the difficult game in the *Test‐Random* condition in Experiment 2. Our work shows that the ability to effectively adapt ecological active practice choices to specific goals and task characteristics may emerge during early childhood. In this sense, young children seem to be equipped with the tools they need to prepare for the unknown.

## Author Contributions

All authors conceived and designed the experiment(s). D.S. piloted the experiment(s) and analyzed the data. D.S. wrote the first draft of the manuscript. All authors interpreted the results and contributed to the manuscript.

## Conflicts of Interest

The authors declare no conflicts of interest.

## Supporting information


Data S1.

## Data Availability

The data and code required to replicate the analyses presented in this study are accessible upon request from the first author. The analyses for Experiments 1 and 2 were preregistered. Additionally, the materials essential for attempting to replicate the reported findings are provided in the Supporting Information.
