# Supplementary material for: Children Strategically Decide What to Practice
Source: Child Dev. 2025 May 31;96(5):1619–31. doi: 10.1111/cdev.14268 (PMC12379865; doi:10.1111/cdev.14268)
Supplement: Supplementary file 1 — Data S1. [file CDEV-96-1619-s001.docx]

Children strategically decide what to practice -

Supplementary material

1

# Experiment 1

We first present a hypothesis rationale that participants in our experiment might have used to decide which game to practice when facing different conditions. We then examine the child and adult samples separately, addressing the manipulation check and comprehension check questions. First, we demonstrate that our statistical analyses remain robust even when excluding rounds where children’s answers during the familiarization phase (i.e., how many items they guessed correctly) deviated from our expectations (e.g., they guessed fewer or more items correctly than 3/4 in the easy game or more items correctly than 0/4 in the difficult game). Second, we show that our statistical analyses remain significant even when including trials where children failed to answer the comprehension check question correctly, in which they had to indicate which of the two games they performed better in. Finally, we provide a comprehensive overview of the analyses performed on both child and adult samples for each round.

## Hypothesis Rationale

In Experiment 1, participants practice choices may have followed the following rationale: In the familiarization phase, participants played an easy game consisting of three easy and one difficult to guess item (we implemented one difficult to guess item in order to make the game easy, but not to easy, see Methods for a detailed explanation) and one difficult game consisting of 4 difficult to guess items. We predicted that after having familiarized themselves with the easy and the difficult game and when facing the *Test-Easy* condition they should practice the easy game, where they scored 3/4 (i.e., 3 out of 4). This would significantly improve their performance at test and allow them to reach the maximum score at test (8/8). If they practiced the difficult game instead, they would achieve a minimum score of 3/8 (the items they had already guessed correctly during the familiarization phase) and a maximum of 7/8, if they were able to guess all the test items. Note that if participants projected the same proportion of easy/difficult-to-guess items in the test as in the familiarization set (3/4, with one difficult item), they should expect to achieve a score of 6/8 on average at test if they trained the difficult game. When facing the *Test-Difficult* condition, participants should practice the difficult game, where they scored 0/4, to increase their likelihood of reaching the maximum score on the test (8/8). If they practiced the easy game instead, they would achieve a minimum average score of 0/8 and a maximum average score of 4/8, if they were able to guess all the test items. Note that if participants projected the same proportion of easy/difficultto-guess items in the test as in the familiarization set (0/4, with 4 difficult items), they should expect to achieve an average score of 0/8 at test if they trained the easy game. When facing the *Test-Random* condition participants should practice the difficult game. In this way, if they were tested in the easy game (50% chance), but had practiced the difficult game they would achieve a minimum score of 3/8 (those they had already guessed during familiarization) and a maximum score of 7/8—or 6/8 if participants projected the same proportion of easy/difficult-to-guess items in the test as in the familiarization set (3/4, with one difficult item); if they were tested in the difficult game (50% chance) and had practiced the difficult game they would achieve the maximum score (8/8) assuming perfect recall of the practiced items. If they practiced the easy game, they would achieve the maximum score 8/8 if tested on the easy game, but a score of 0 if tested on the difficult game.

# Child Sample

## Manipulation Check

We checked whether we successfully manipulated game difficulties. We expected children to guess 3/4 items correctly in the easy game, and 0/4 items correctly in the difficult game (see Table 1 and and Table 2. In the analyses we report in the main paper we included children regardless of whether they performed the familiarization phase as we expected. Note that this inclusion had no effect on the statistical results we found.

Table 1: Children’s Cumulative Performance in the Easy Game Familiarization Across Three Rounds

| Items Guessed | 0/4 | 1/4 | 2/4 | 3/4 | 4/4 |
| --- | --- | --- | --- | --- | --- |
| Count | 0 | 4 | 30 | 299 | 12 |
| Percentage (%) | 0 | 1.16 | 8.80 | 86.67 | 3.48 |

Table 2: Children’s Cumulative Performance in the Difficult Game Familiarization Across Three Rounds

| Items Guessed | 0/4 | 1/4 | 2/4 | 3/4 | 4/4 |
| --- | --- | --- | --- | --- | --- |
| Count | 330 | 15 | 0 | 0 | 0 |
| Percentage (%) | 95.65 | 4.35 | 0 | 0 | 0 |

## Comprehension Check

After children played the easy and the difficult game, they performed a comprehension check in which they had to indicate the game at which they were better. Participants successfully completed the comprehension check in 238 out of 245 rounds (97%). In the analyses of the main paper we excluded 7 rounds in which participants failed to answer the comprehension check correctly. This removal had no effect on the statistical results we found.

## Children’s Practice Choices by Round

Round 1

We analyzed children’s practice choices in round 1. A logistic mixed-effects model predicting children’s practice choices (easy or difficult) with condition (*Test-Easy, Test-Difficult, Test-Random; Test-Easy* as baseline) and age in months as fixed effects and participants’ ID as a random effect revealed main effects of the *Test-Difficult* condition (*p* = *.*028, *OR* = 0*.*314 [0.108 – 0.855]) the *Test-Random* condition (*p* = *.*014, *OR* = 0*.*289 [0.103 – 0.759]), and age in months (*p* = *.*002, *OR* = 0*.*506 [0.318 – 0.772]).

Looking at the interaction effects of the interaction of condition and age revealed no significant effects (*Test-Difficult* condition and age in months: *p* = *.*264, *OR* = 0*.*524 [0.147 – 1.528]; *TestRandom* condition and age in months: *p* = *.*840, *OR* = 0*.*895 [0.292 – 2.609]).

Round 2

Next, we analyzed children practice choices in round 2. A logistic mixed-effects model predicting children’s practice choices (easy or difficult) with condition (*Test-Easy, Test-Difficult, TestRandom; Test-Easy* as baseline) and age in months as fixed effects and participants’ ID as a random effect revealed main effects of the *Test-Difficult* condition (*p* = *.*001, *OR* = 0*.*163 [0.052 – 0.466]) the *Test-Random* condition (*p* = *.*089, *OR* = 0*.*449 [0.175 – 1.120]), but no effect of age in months (*p* = *.*459, *OR* = 0*.*859 [0.571 – 1.282]).

Looking at the interaction effects of conditions and age revealed a significant effect of the interaction of condition and age (*Test-Difficult* condition and age in months: *p* ¡ .007, *OR* = 0.140 [0.029 – 0.530]; *Test-Random* condition and age in months: *p* = .010, *OR* = 0.256 [0.084 – 0.691]).

Round 3

Next, we analyzed children practice choices in round 3. A logistic mixed-effects model predicting children’s practice choices (easy or difficult) with condition (*Test-Easy, Test-Difficult, TestRandom; Test-Easy* as baseline) and age in months as fixed effects and participants’ ID as a random effect revealed main effects of the *Test-Difficult* condition (*p* = *.*002, *OR* = 0*.*200 [0.069 – 0.534]) the *Test-Random* condition (*p < .*001, *OR* = 0*.*106 [0.030 – 0.033]), and age in months (*p* = *.*002, *OR* = 0*.*475 [0.290 – 0.748]).

Looking at the interaction effects of conditions and age revealed a significant effect of the interaction of condition and age (*Test-Difficult* condition and age in months: *p* = .007, *OR* = 0.902 [0.831 – 0.967]; *Test-Random* condition and age in months: *p* = .054, *OR* = 0.923 [0.839 – 0.994]).

## Children’s Practice Choice by Age Group

Table 3: Children’s Active Practice Choice by Age Group

| Age in Years | N Adaptive Practice | N per Age Group | Percent (%) |
| --- | --- | --- | --- |
| 4 | 39 | 71 | 54.93 |
| 5 | 37 | 70 | 52.86 |
| 6 | 43 | 65 | 66.15 |
| 7 | 53 | 66 | 80.30 |
| 8 | 54 | 66 | 81.81 |

## Children’s Test Scores

Overall performance

Table 4: Average number of points achieved by children in different conditions and age groups over three rounds

Age in Years

| Condition | 4 | 5 | 6 | 7 | 8 |
| --- | --- | --- | --- | --- | --- |
| *Test-Easy* | 7.38 | 7.00 | 7.55 | 7.46 | 7.59 |
| *Test-Difficult* | 2.17 | 2.57 | 4.91 | 6.00 | 6.09 |
| *Test-Random* | 2.44 | 1.71 | 3.41 | 5.36 | 5.64 |

Round 1

Table 5: Average number of points achieved by children in different conditions and age groups in Round 1.

Age in Years

| Condition | 4 | 5 | 6 | 7 | 8 |
| --- | --- | --- | --- | --- | --- |
| *Test-Easy* | 7.66 | 7.00 | 7.57 | 7.18 | 7.46 |
| *Test-Difficult* | 2.09 | 3.43 | 3.00 | 5.00 | 6.57 |
| *Test-Random* | 3.57 | 1.13 | 3.70 | 6.14 | 4.25 |

Round 2

Table 6: Average number of points achieved by children in different conditions and age groups in Round 2.

Age in Years

| Condition | 4 | 5 | 6 | 7 | 8 |
| --- | --- | --- | --- | --- | --- |
| *Test-Easy* | 7.38 | 7.25 | 7.80 | 7.62 | 8.00 |
| *Test-Difficult* | 4.33 | 3.00 | 6.44 | 6.88 | 5.5 |
| *Test-Random* | 2.25 | 1.00 | 2.50 | 4.17 | 5.70 |

Round 3

Table 7: Average number of points achieved by children in different conditions and age groups in Round 3.

Age in Years

| Condition | 4 | 5 | 6 | 7 | 8 |
| --- | --- | --- | --- | --- | --- |
| *Test-Easy* | 7.20 | 6.71 | 7.40 | 8.00 | 7.57 |
| *Test-Difficult* | 1.56 | 1.38 | 4.12 | 5.70 | 6.29 |
| *Test-Random* | 1.00 | 3.00 | 4.50 | 5.56 | 6.25 |

## 0.1 Practice choices by media split

To further investigate the observed age effects, we performed exploratory analyses within a younger and older age group via a median split by age (cutoff at 75 months; age 6.25 years). A logistic mixed-effects model predicting practice choices (easy or difficult) in the older age group with condition (*Test-Test-Easy, Test-Test-Difficult, Test-Test-Random; Test-Test-Easy* as baseline) and age in months as fixed effects and participants’ ID as a random effect revealed a significant effect of the *Test-Test-Difficult* condition (*p < .*001, *OR* = 0*.*044 [0.012 – 0.161]) and the *Test-TestRandom* condition (*p < .*001, *OR* = 0*.*125 [0.046 – 0.344]), but not age in months (*p* = *.*195, *OR* = 0*.*762 [0.505 – 1.150]). In the older age group, 36% of the children (*n* = 19/53) selected the difficult game in the *Test-Test-Easy* condition (significantly lower than chance; *p* = *.*026; two-tailed 50% binomial test). In the *Test-Test-Difficult* condition and *Test-Test-Random* condition 93% (*n* = 49/53) and 81% (*n* = 43/53) of the older children, respectively, selected the difficult game. Both proportions were significantly greater than chance (*Test-Test-Difficult* condition: *p < .*001; *Test-Test-Random* condition: *p < .*001, two-tailed 50% binomial test). A chi-square test revealed no difference in task choice between the *Test-Test-Difficult* and *Test-Test-Random* conditions (*χ*^2^(1) = 2*.*058, *p* = *.*151). The same model did not reveal any significant main effects of condition or age in the younger age group (see Fig. 1).

## Retrospective Evaluation

After each test, we reminded children of their practice choices during the practice phase and asked whether they believed their choices were a good idea (yes or no). We introduced a binary dummy variable to assess the alignment between their practice decisions and their retrospective evaluations. We assigned a value of 1 to cases where participants made adaptive practice choices (e.g., practicing the easy game in the *Test-Easy* condition, or the difficult game in the *Test-Difficult* and *Test-Random* conditions) and indicated that these choices were retrospectively beneficial. Similarly, a value of 1 was assigned if participants made non-adaptive practice decisions and retrospectively indicated them as unfavorable. In all other cases, when there was no alignment between practice decisions and retrospective evaluations, we assigned a value of 0.

A logistic mixed-effects model, predicting a match between practice choices and their retrospective evaluation (coded as 0/1) by condition, age in months, and their interaction as fixed effects, and participants’ ID as a random effect revealed a significant interaction interaction effect of the *Test-Difficult* condition and age (*p* = *.*005, *OR* = 1*.*095 [1.027 – 1.167]); as well as the *Test-Random* condition and age (*p* = *.*004, *OR* = 1*.*089 [1.028 – 1.153]). Indicating that with age children in both condition were more accurate in retrospectively evaluating the practice choices they had made.

# Adult Sample

## Manipulation Check

We first checked whether we successfully manipulated game difficulties (in the easy game participants should guess 3/4 items, in the difficult game they should guess 0/4 items; see Table 8 and Table ??).

Table 8: Comparison of adults’ performance in the familiarization with the easy game

| Items Guessed | 0/4 | 1/4 | 2/4 | 3/4 | 4/4 |
| --- | --- | --- | --- | --- | --- |
| Count | 0 | 0 | 7 | 80 | 6 |
| Percentage (%) | 0 | 0 | 7.53 | 86.02 | 6.45 |

Table 9: Comparison of adults’ performance in the familiarization with the difficult game

| Items Guessed | 0/4 | 1/4 | 2/4 | 3/4 | 4/4 |
| --- | --- | --- | --- | --- | --- |
| Count | 73 | 19 | 0 | 1 | 0 |
| Percentage (%) | 78.49 | 20.43 | 0 | 1.07 | 0 |

## Comprehension Check

After participants played each game they performed a comprehension check in which they had to indicate the game in which they were better at. All participants correctly indicated the easy game. All participants answered the comprehension check questions correctly.

## Adult’s Practice Choices by Round

Round 1

A logistic mixed-effects model predicting participants’ practice choices (easy or difficult) with condition (*Test-Easy, Test-Difficult, Test-Random; Test-Easy* as baseline) and age in years as fixed effects and participants’ ID as a random effect revealed main effects of the *Test-Difficult* condition (*p < .*001, *OR* = 0*.*032 [0.005 – 0.208]) and the *Test-Random* condition (*p < .*001, *OR* = 0*.*018 [0.003 – 0.116]).

Round 2

We first looked at adults’ behavior in round 2. A logistic mixed-effects model predicting participants’ practice choices (easy or difficult) with condition (*Test-Easy, Test-Difficult, Test-Random; Test-Easy* as baseline) and age in years as fixed effects and participants’ ID as a random effect revealed main effects of the *Test-Difficult* condition (*p* = *.*001, *OR* = 0*.*625 [0.266 – 0.984]) and the *Test-Random* condition (*p < .*001, *OR* = 0*.*769 [0.451 – 1.088]).


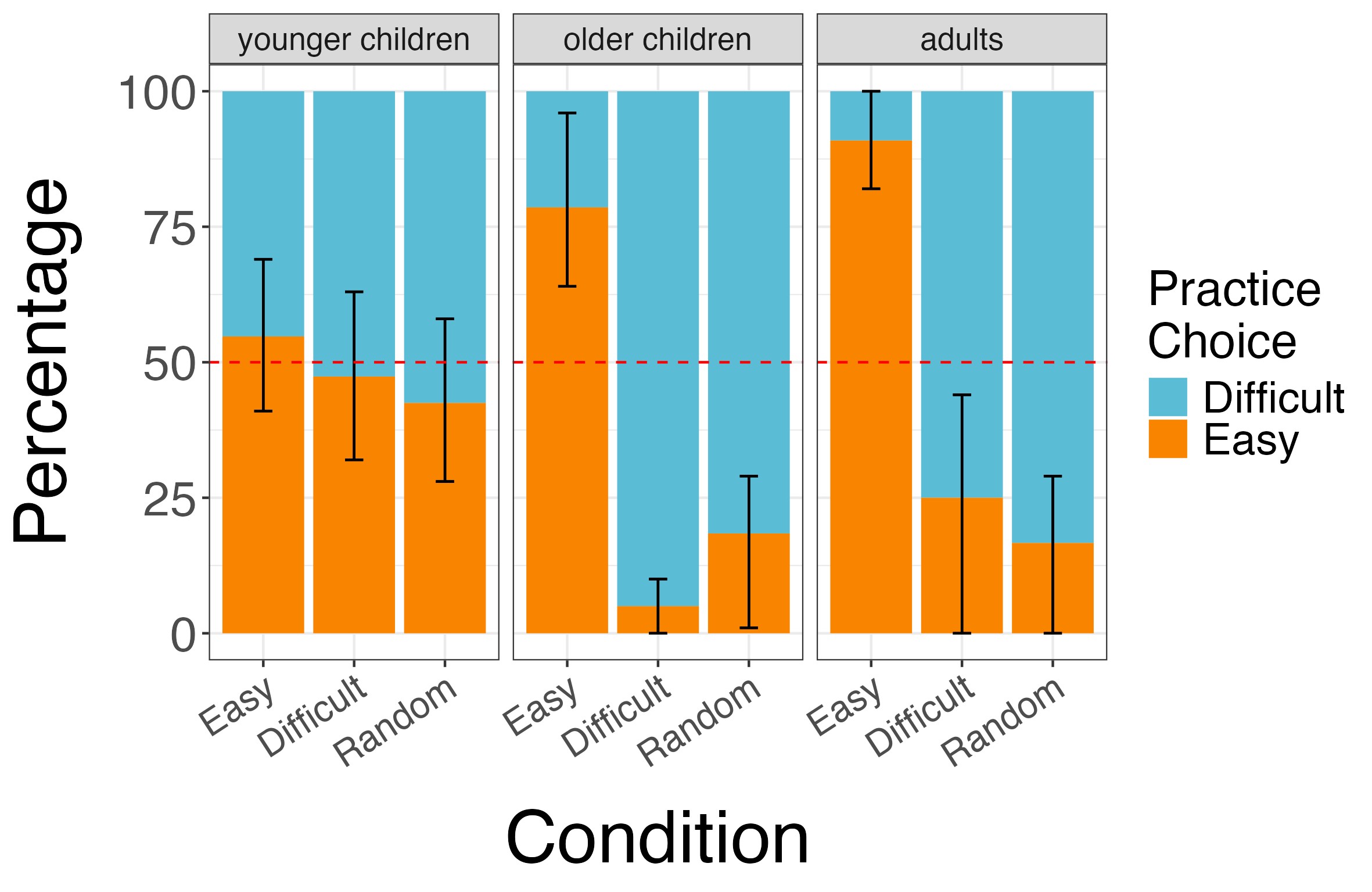


Figure 1: Illustration of participants’ practice choices by condition in Experiment 1. Participants’ practice choices, displayed by age group (young children vs old children (cutoff at 75 months) vs adults) and condition. Bars represent *the Easy, Difficult*, and *Random* conditions. The red dotted line indicates the 50% chance level. The error bars extending above and below the bars represent the 95% confidence intervals, indicating the range within which the true population means are likely to fall.

Round 3

Next, we analyzed participants practice choices in round 3. A logistic mixed-effects model predicting participants’ practice choices (easy or difficult) with condition (*Test-Easy, Test-Difficult, Test-Random; Test-Easy* as baseline) and age in years as fixed effects and participants’ ID as a random effect revealed main effects of the *Test-Difficult* condition (*p* = *.*007, *OR* = 0*.*285 [0.001 – 0.273]) and the *Test-Random* condition (*p* = *.*003, *OR* = 0*.*020 [0.001 – 0.182]).

## Retrospective Evaluation

We introduced the same binary dummy variable to assess the alignment between practice decisions and participants’ retrospective evaluations as in the child sample. A logistic mixed-effects model, predicting a match between adults’ practice choices and their retrospective evaluation (coded as 0/1) by condition as a fixed effect, and participants’ ID as a random effect revealed no significant effects.

# Experiment 2

## Manipulation Check

We first analyzed how much time it took children to build the easy and the difficult tower during familiarization phase and whether these times differed significantly between the easy and the difficult games. As expected, children needed less time to build the easy tower (*M* = 8*.*47*seconds*, *SD* = 4*.*38) compared to building the difficult tower (*M* = 20*.*58*seconds* , *SD* = 16*.*57; *t*(81.985) = -6.036, *p <* 0*.*001, Welch two-sample *t*-test).

## Comprehension Check

We excluded a total 13 children because they failed to answer one or multiple of the comprehension check question correctly even after we repeated the relevant part of the procedure with them for a maximum of three times. In particular, we excluded 7 children because they claimed to know in which game they would be tested in the *Test-Random* condition, even after demonstrating the procedure to them several times. We excluded 6 children who failed to indicate the easy game as easy and the difficult game as difficult.

## Retrospective Evaluation

We introduced the same binary dummy variable to assess the alignment between practice decisions and participants’ retrospective evaluations as in Experiment 1.

A logistic mixed-effects model, predicting a match between practice choices and children’s retrospective evaluation (coded as 0/1) by condition revealed no significant effects.
